# Supplementary material for: Circulating transforming growth factor-β1 facilitates remyelination in the adult central nervous system
Source: eLife. 2019 May 9;8:e41869. doi: 10.7554/eLife.41869 (PMC6508935; doi:10.7554/eLife.41869)
Supplement: Figure 1—source data 1. — Inhibitors were used at a final concentration of 10 μM. Each line shows the relative values of MBP/Olig2 area (two rows from the left) and Olig2 cell number (two rows from the right) relative to the control value (DMSO treatment without serum). TGF-βRI kinase inhibitor (red letters) is the only drug we tested that targets a receptor-type protein. The data is average of two trials. [file elife-41869-fig1-data1.pdf]

Figure 1-source data 1

Product Description	Relative area of MBP/Olig2		Relative cell number of Olig2	
	Control	Serum	Control	Serum
Control (DMSO)	1	4.075038	1	1.540595
DNA-PK Inhibitor II	1.072964	0.816265	1.205717	1.965316
GSK-3 Inhibitor X	1.553935	0.841329	0.98298	2.020891
Syk Inhibitor	1.116218	1.040838	0.697921	2.024712
ATM/ATR Kinase Inhibitor	1.150339	0.793869	1.116171	2.040537
DNA-PK Inhibitor III	1.724457	1.241198	0.954285	2.157534
GSK-3β Inhibitor XI	1.35744	1.646938	1.060676	1.856841
p38 MAP Kinase Inhibitor	1.431496	1.580869	0.980869	2.014143
Purvalanol A	1.652472	1.343381	0.982004	1.571936
PD98059	1.140504	1.875568	0.877135	2.078993
Syk Inhibitor III	0.900556	1.563802	0.861825	1.738104
PD169316	1.469447	1.985862	1.147018	1.384028
TGF-βRI Kinase Inhibitor	0.551418	1.363995	0.985175	2.081429
Sphingosine Kinase Inhibitor	0.880749	1.983994	0.663828	1.03917
MEK Inhibitor II	0.756446	1.835005	1.054865	1.879065
MNK1 Inhibitor	0.724727	0.899254	0.871254	1.12726
GSK-3β Inhibitor VIII	1.910861	1.688785	0.594019	1.163565
PKCβII/EGFR Inhibitor	0.527494	1.699848	0.54238	1.659975
